# Radiotherapy quality assurance in the TROG 12.01 randomised trial and its impact on loco-regional failure

**DOI:** 10.3389/fonc.2023.1333098

**Published:** 2024-02-05

**Authors:** June Corry, Alisha Moore, Liz Kenny, Chris Wratten, Tsien Fua, Charles Lin, Sandro Porceddu, Chen Liu, Michael Ruemelin, Amy Sharkey, Lachlan McDowell, Dean Wilkinson, Albert Tiong, Danny Rischin

**Affiliations:** ^1^ Genesiscare Radiation Oncology Department, St Vincents Hospital, Melbourne, VIC, Australia; ^2^ Department Medicine, University of Melbourne, Melbourne, VIC, Australia; ^3^ Department Radiation Oncology, Peter MacCallum Cancer Center, Melbourne, VIC, Australia; ^4^ Department Radiation Quality Assurance, Trans-Tasman Radiation Oncology Group (TROG), Newcastle, NSW, Australia; ^5^ Department Radiation Oncology, Royal Brisbane and Women’s Hospital, Brisbane, QLD, Australia; ^6^ Faculty Medicine, University of Queensland, Brisbane, QLD, Australia; ^7^ Department Radiation Oncology, Calvary Mater Hospital and University Newcastle, Newcastle, NSW, Australia; ^8^ Department Radiation Oncology, Princess Alexander Hospital, Brisbane, QLD, Australia; ^9^ Department Radiation Therapy, Peter MacCallum Cancer Center, Melbourne, VIC, Australia; ^10^ Department Radiation Therapy, Illawarra Cancer Care Centre, Wollongong, NSW, Australia; ^11^ Department Medical Oncology, Peter MacCallum Cancer Center, Melbourne, VIC, Australia; ^12^ Sir Peter MacCallum Department of Oncology, University of Melbourne, Melbourne, VIC, Australia

**Keywords:** quality assurance, intensity modulated radiotherapy (IMRT), radiotherapy, head and neck (H&N) cancer, human papilloma virus - HPV

## Abstract

**Introduction:**

There is no consensus as to what specifically constitutes head and neck cancer radiotherapy quality assurance (HNC RT QA). The aims of this study are to (1) describe the RT QA processes used in the TROG 12.01 study, (2) review the RT QA processes undertaken for all patients with loco-regional failure (LRF), and (3) provide prospective data to propose a consensus statement regarding the minimal components and optimal timing of HNC RT QA.

**Materials and methods:**

All patients undergoing RT QA in the original TROG 12.01 study were included in this substudy. All participating sites completed IMRT credentialling and a clinical benchmark case. Real-time (pre-treatment) RT QA was performed for the first patient of each treating radiation oncologist, and for one in five of subsequent patients. Protocol violations were deemed major if they related to contour and/or dose of gross tumour volume (GTV), high dose planning target volume (PTVhd), or critical organs of risk (spinal cord, mandible, and brachial plexus).

**Results:**

Thirty HNROs from 15 institutions accrued 182 patients. There were 28 clinical benchmark cases, 27 pre-treatment RT QA cases, and 38 post-treatment cases. Comprehensive RT QA was performed in 65/182 (36%) treated patients. Major protocol violations were found in 5/28 benchmark cases, 5/27 pre-treatment cases, and 6/38 post-treatment cases. An independent review of all nine LRF cases showed major protocol violations in four of nine cases.

**Conclusion:**

Only pre-treatment RT QA can improve patient outcomes. The minimal components of RT QA in HNC are GTVs, PTVhd, and critical organs at risk. What constitutes major dosimetric violations needs to be harmonised.

## Introduction

Since the publication of the landmark study by Peters et al. (1), head and neck radiation oncologists (HNROs) have been aware of the importance of the quality of radiotherapy in optimising patients’ loco-regional control and overall survival. Since that study, some form of radiotherapy quality assurance (RT QA) has been incorporated into the majority of head and neck cancer (HNC) clinical trials.

However, the term “radiotherapy quality assurance” is an umbrella term with no current consensus as to its optimal components, the timing, or what is the optimal percentage of HNC patients who should undergo RT QA.

We have previously retrospectively shown the impact of pre-treatment RT QA of all curative intent cases at a large HNC centre (2). In our study the RT QA consisted of a review of the staging imaging and of the gross tumour volumes (GTVs), planning treatment volumes (PTVs), and critical organs at risk (OARs) by a second RO for *all* patients having curative intent non-surgical treatment. This RT QA occurred prior to dosimetric planning and prior to treatment.

While most HNC published studies include a statement regarding performance of RT QA in their study, the precise details of that RT QA are not always included. There is general agreement in HNC that a major goal of RT QA is to reduce errors that are likely to lead to reduced tumour control probability and/or significant and serious treatment-related toxicity. Questions remain as to what is the optimal RT QA process required to achieve this goal—how do we balance effectiveness, efficiency, and cost within the RT QA process? Clearly, real-time review of every case would be optimal, but this is seen as costly in terms of time and effort and possibly unnecessary.

This prospective study reports fully on the RT QA processes that were used in the randomised trial of weekly cetuximab versus weekly cisplatin and radiation in good prognosis loco-regionally advanced HPV-associated oropharyngeal squamous cell carcinoma—TROG 12.01 (3).

The aims of this study are to (1) give a detailed account of the RT QA processes used in the original study, (2) review the RT QA processes that had been undertaken for all patients who failed loco-regionally, and (3) provide prospective data to propose a consensus statement regarding the minimal components and optimal timing of HNC RT QA.

## Methods

**Figure 1:**
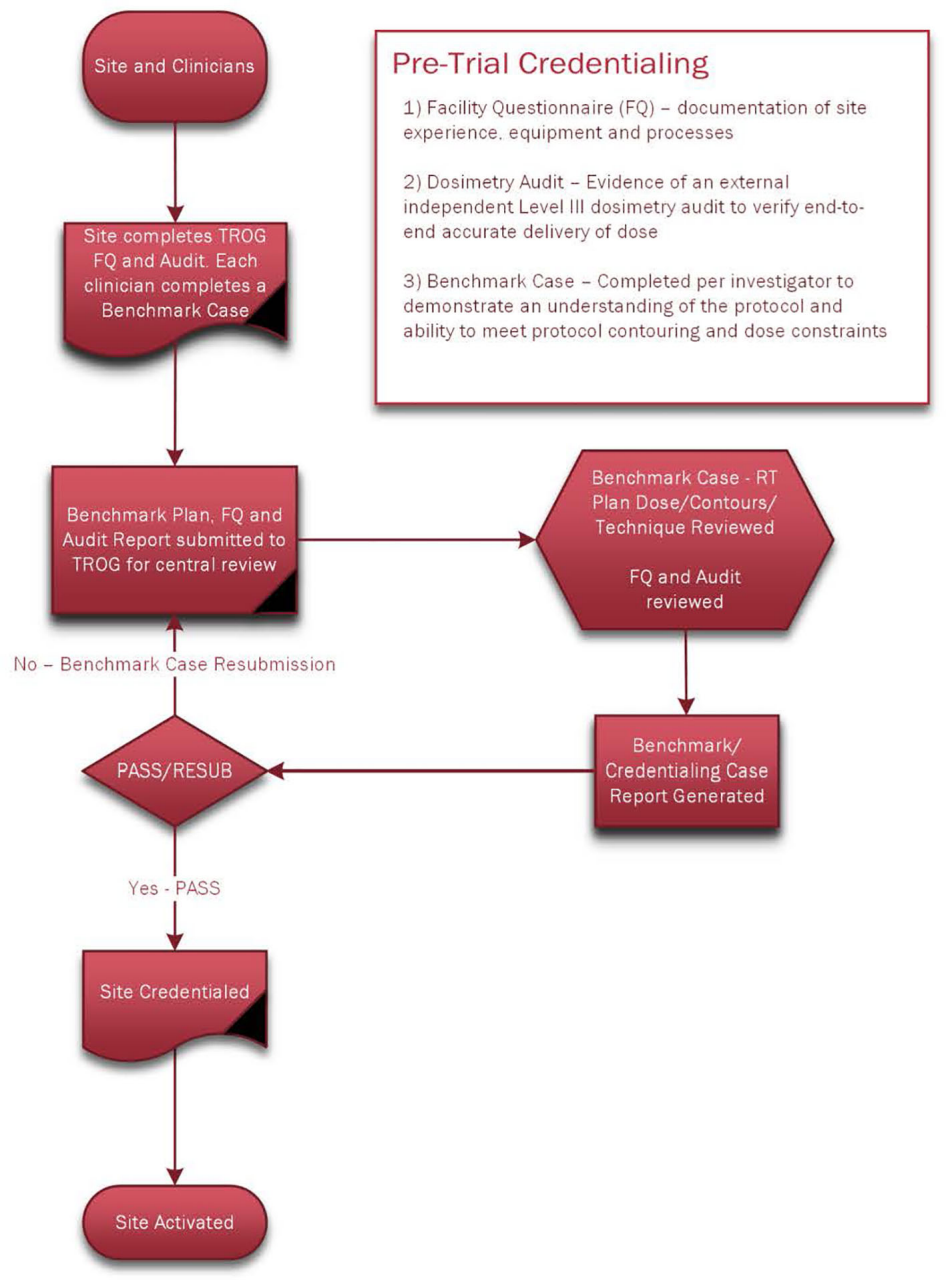
Pre-Trial Credentialing.

Intensity-modulated radiotherapy (IMRT) was mandatory for participation in the TROG 12.01 study. All participating sites in TROG 12.01 had pre-study credentialing that included completion of a volumetric arc therapy (VMAT)/IMRT facility questionnaire, a Level III dosimetry audit (determining the absorbed dose delivered to selected points within an anthropomorphic phantom; this is an end-to-end audit where the phantom undergoes all steps within the radiotherapy treatment chain), and submission of a library benchmarking case for RT QA review of dosimetry and of all protocol contours by an independent HNRO from a panel of five HNROs. Once the library benchmark case was successfully completed, sites were then eligible to commence patient accrual (**Figure 1**).

In centres where one or more benchmark case had been submitted by other HNROs from that centre, subsequent benchmarking cases were not required for the remaining HNROs enrolling patients from that centre on the proviso that they had a robust institutional RT QA programme (i.e., defined as a review of all new definitive cases’ imaging and contours) and their first case underwent real-time RT QA prior to commencing treatment.

**Figure 2:**
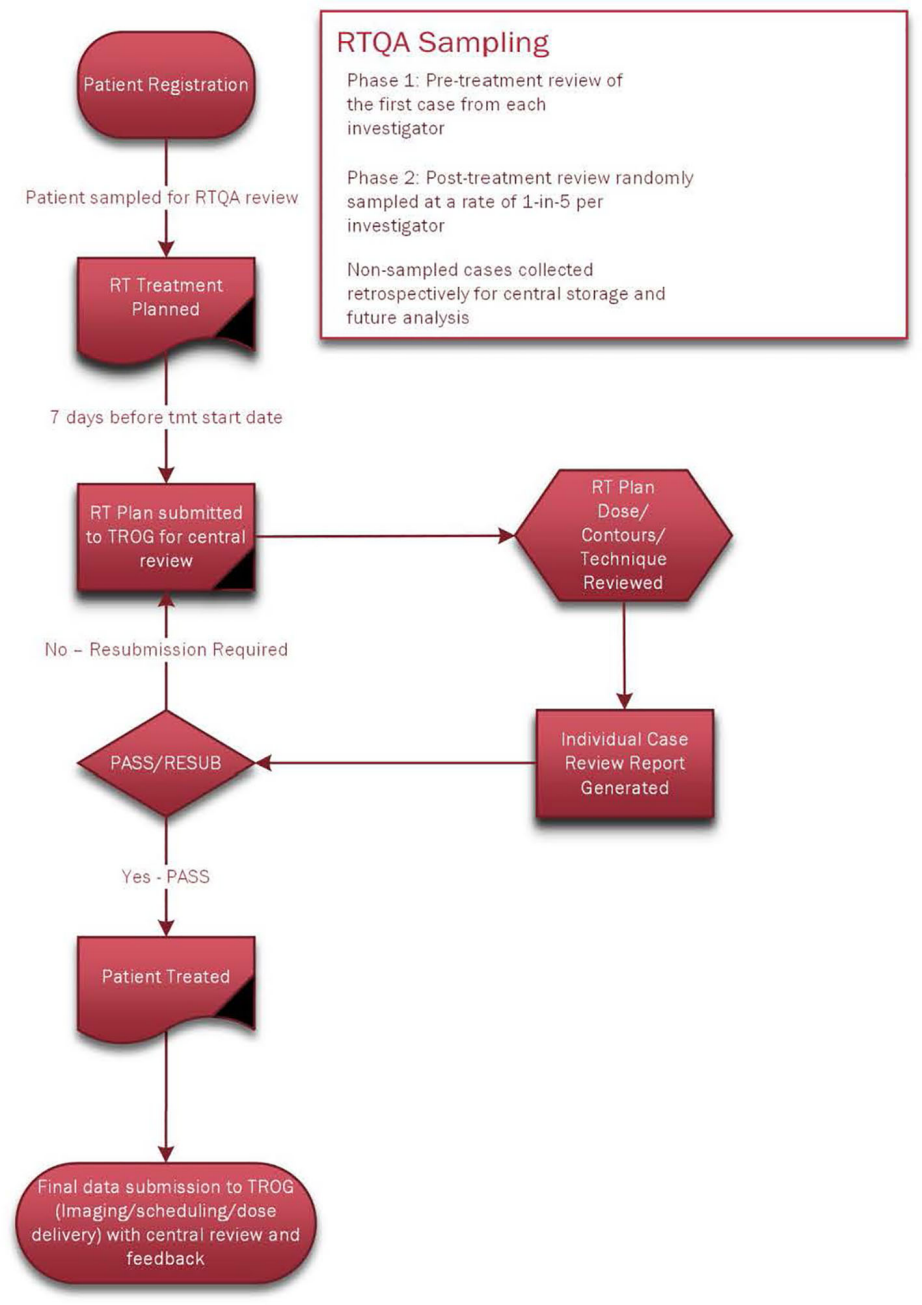
On-Trial Radiotherapy Quality Assurance.

A central real-time pre-treatment RT QA review was performed for the first patient of each radiation oncologist investigator, and post-treatment reviews were performed for one in five patients for each investigator (**Figure 2**).

Centres sent the diagnostic images and the simulation CT images with all radiotherapy dosimetry and contouring data to the Trans-Tasman Oncology Group (TROG) group. Dosimetry, treatment delivery, and scheduling were reviewed centrally, and the accuracy of all the contouring volumes was reviewed by an independent member of the five-HNRO review panel. Each panel member specialised in HNC and had five or more years of experience.

Each investigator had a copy of the protocol that detailed the RT techniques, including GTV to PTV margins of minimum 10 mm, and the criteria for unilateral RT. In addition, each investigator had the table of items to be reviewed and the acceptable, minor, and major protocol violations for each of these structures (**Table 1**).

**Table 1:** Major and minor protocol violations criteria in TROG 12.01.

Question	Protocol	Baseline	Acceptable	Minor variation	Major variation	Missing/Invaluable
Target Volumes—GTV & CTV						
GTV-P: Minimum Dose (D100%)	No	>/=66.5 Gy	N/A	N/A	N/A	N/A
Percentage GTV-P receiving <95% of the prescribed dose (V95)	Yes	0	0%–2%	2.01%–7%	>7%	N/A
GTV-N: Minimum Dose (D100%)	No	>/=66.5 Gy	N/A	N/A	N/A	N/A
Percentage GTV-N receiving <95% of the prescribed dose (V95)	Yes	0	0%–2%	2.01%–7%	>7%	N/A
Is the maximum dose contained within the CTV?	No	Yes	Yes	N/A	N/A	N/A
Target Volumes—PTV						
PTV70Gy: D95%	Yes	>/=66.5 Gy	>/=66.5 Gy	65.1–66.49 Gy	<65.1 Gy	N/A
Percentage of PTV70 receiving </=66.5 Gy (V95%)	Yes	0	0%–5%	5.01%–7%	>7%	N/A
PTV70: Near Minimum (D98%)	No	>/=66.5 Gy	N/A	N/A	N/A	N/A
PTV70: Median Dose (D50%)	No	70 Gy	68.6–71.4 Gy	N/A	N/A	N/A
PTV70: Near Maximum (D2%)	Yes	70 Gy	<110% (76.99 Gy)	110%–115% (77–80.5 Gy)	>115% (80.51 Gy)	N/A
PTV67Gy: D95%	Yes	>/=63.65 Gy	>/=63.65 Gy	60.3–63.64 Gy	<60.3 Gy	N/A
Percentage of PTV67 receiving </=63.65 Gy (V95%)	Yes	0	0-5%	5.01%–10%	>10%	N/A
PTV67: Near Minimum (D98%)	No	>/=63.65 Gy	N/A	N/A	N/A	N/A
PTV63Gy: D95%	Yes	>/=59.85 Gy	>/=59.85 Gy	58.6–59.84 Gy	<58.6 Gy	N/A
Percentage of PTV63 receiving </=59.85 Gy (V95%)	Yes	0	0%–5%	5.01%–10%	>10%	N/A
PTV63: Near Minimum (D98%)	No	>/=59.85 Gy	N/A	N/A	N/A	N/A
PTV54Gy: D95%	Yes	>/=51.3 Gy	>/=51.3 Gy	45.9–51.29 Gy	<45.9 Gy	N/A
Percentage of PTV54 receiving </=51.3 Gy (V95%)	Yes	0	0%–5%	5.01%–15%	>15%	N/A
PTV54: Near Minimum (D98%)	No	>/=51.3 Gy	N/A	N/A	N/A	N/A
Critical OARs						
Spinal Cord: D1%	Yes	</=45 Gy	</=45 Gy	0%–3% (45.01–46.35 Gy)	>3% (>46.35 Gy)	N/A
Spinal Cord: Maximum Point Dose	No	</=45 Gy	N/A	N/A	N/A	N/A
Spinal Cord: PRV (Sc + 5 mm): D1%	Yes	</=50 Gy	</=50 Gy	0%–3% (50.01–51.5 Gy)	6(>51.5 Gy)	N/A
Spinal Cord: PRV (Sc + 5 mm): Maximum Point Dose	No	</=50 Gy	N/A	N/A	N/A	N/A
Brachial Plexus Left: D1%	Yes	</=66 Gy	</=66 Gy	0%–3% (66.01–67.98Gy)	>3% (>67.98Gy)	N/A
Brachial Plexus Left: Maximum Point Dose	No	</=66 Gy	N/A	N/A	N/A	N/A
Brachial Plexus Right: D1%		</=66 Gy	</=66 Gy	0-3% (66.01–67.98Gy)	>3% (>67.98Gy)	N/A
Brachial Plexus Right: Maximum Point Dose	No	</=66 Gy	N/A	N/A	N/A	N/A
Other OARs						
Mandible D1%	Yes	</=70 Gy	</=70 Gy	0-3% (70.01–72.1 Gy)	>3% (>72.1 Gy)	N/A
Mandible: Maximum Point Dose	No	</=70 Gy	N/A	N/A	N/A	N/A
Parotid Gland (Right): Mean Dose	No	</=26 Gy	</=26 Gy	N/A	N/A	N/A
Parotid Gland (Left): Mean Dose	No	</=26 Gy	</=26 Gy	N/A	N/A	N/A
Glottic Larynx: Mean Dose	No	</=45 Gy	</=45 Gy	N/A	N/A	N/A
Constrictors: Mean Dose	No	</=63 Gy	</=63 Gy	N/A	N/A	N/A
Oral Cavity: Mean Dose	No	</=42 Gy	</=42 Gy	N/A	N/A	N/A

Major protocol violations were those with major variations in contour and/or dose to the Priority 1 structures (GTVp, GTVn, and PTV70 and critical organs at risk—spinal cord, mandible, and brachial plexus). All other structures were placed in the Priority 2 category (PTV intermediate and low dose, pharyngeal constrictors, larynx, parotids, and oral cavity). The protocol included contouring atlases for brachial plexus and pharyngeal constrictors.

### Statistical analysis

Formal statistical analysis was not required. This report is a presentation of the RT QA results and was limited to descriptive reporting and percentages.

All cases of loco-regional failure (LRF) were reviewed by one of the authors (JC). These cases were blinded for institutional site and responsible radiation oncologist; the actual site of failure (i.e., either local or regional, or both) and the clinical outcomes for each case were also unknown. Once the RT QA review was complete for that list of patients, then the site of failure was made known so that a correlation could then be made between any major protocol violations and the clinical likelihood of that violation (RT dose and/or contour) contributing to the site of failure. For each case, it was also documented what prospective RT QA had been performed in relation to that case, specifically whether a benchmark case had been submitted, and whether pre-treatment or post-treatment RT QA had been performed. We also documented the presence or absence of institutional RT QA for each case.

## Results

Overall, there were 182 patients available for analysis in the TROG 12.01 study. All except one patient from each arm (180/182, 99%) received the prescribed dose in the prescribed time frame, 70 Gy in 35 fractions over 7 weeks.

Thirty HNROs from 15 institutions accrued patients to TROG 12.01.

There were 28 library benchmark cases, 27 cases underwent real-time RT QA pre-treatment, and 38 cases had their RT QA review performed after completion of their treatment. Thus, in total, there was comprehensive RT QA performed in 93 cases and in 65/182 (36%) treated patients.

### Benchmark cases

There were 28 benchmark cases submitted. Twenty-three cases were protocol compliant, and five cases required resubmission due to major protocol variations in Priority 1 structures (contouring of brachial plexus 2, contour PTV70, and D1% dose to brachial plexus × 4 and spinal cord × 1). Of these, two were corrected by the responsible HNRO and resubmitted and passed RT QA (2/5, 40%), and one was not resubmitted but the first patient from that investigator was reviewed in real time (thus included in the pre-treatment review section). Two were never resubmitted, and these two investigators did not then participate in the study.

### Pre-treatment review

#### Priority 1 structures

There were 30 clinicians and, thus, 30 patients were to have real-time pre-treatment review. However, two clinicians did not submit their cases with adequate time for pre-treatment review to be completed (and they were subsequently reviewed post-treatment), and one case was missed from pre-treatment review. Thus, there was a total of 27 real-time pre-treatment RT QA review completed. In these 27 cases, there were six major variations in Priority 1 structures in five patients (four in contouring—GTVp contour × 2, PTV70 contour, and brachial plexus contour, and two in dosimetry to the brachial plexus) (D1% > 68 Gy).

There were 13 minor variations in Priority 1 structures in nine patients (7 in contouring—spinal cord × 2, PTV70 × 3, and brachial plexus × 2, and 6 in dosimetry—mandible D1% × 2, brachial plexus D1% × 1, 95% of PTV70 received less than 65.1 Gy × 2, and 100% GTVn receiving less than 66.5 Gy × 1).

#### Priority 2 structures

There were two major variations in two patients in Priority 2 structures (one contour PTV63, one dosimetry 95% of PTV63 received < 58.6 Gy).

There were 20 minor variations in 13 patients in Priority 2 structures (19 in contouring—pharyngeal constrictors × 5, oral cavity × 5, larynx × 2, PTV54 × 3, and PTV63 × 4, and 1 in dosimetry 95% PTV54 receiving less than 51.3 Gy).

Pre-treatment cases with major violations in Priority 1 structures were corrected and resubmitted in three of the five cases. The violations corrected in these resubmitted cases were the GTV contour × 2 and PTV70 contour × 1. The three patients where correction and resubmission were not requested had major violations, namely, contouring of the brachial plexus × 1, and dose to the brachial plexus exceeding 68 Gy × 2.

### Post-treatment review

#### Priority 1 structures

In the 38 cases reviewed post-treatment, there were seven major variations in Priority 1 structures in six patients: two in contouring (GTVn and PTV70) and five in dosimetry (95% of PTV70 receiving less than 65.1 Gy × 2, brachial plexus D1% > 68 Gy, mandible D1%, and 100% GTVp receiving < 66.5 Gy).

There were 24 minor violations in Priority 1 structures in 15 patients: 16 in contouring (GTVp × 1, GTVn × 2, brachial plexus × 8, and spinal cord × 5) and 8 in dosimetry (95% of PTV70 received < 66.5 Gy × 4, mandible D1% × 2, and brachial plexus D1% × 2).

#### Priority 2 structures

There were five major variations in Priority 2 structures in four patients: four in contouring (parotid × 2, pharyngeal constrictors × 1, and PTV54 × 1) and one in dosimetry, 95% PTV54 received < 45.9 Gy).

There were 39 minor variations in Priority 2 structures in 24 patients: 33 in contouring (PTV54 × 12, PTV 63 × 1, pharyngeal constrictors × 7, oral cavity × 9, and larynx × 4) and 6 in dosimetry (95% of PTV67 received < 60.3 Gy × 2, 95% of PTV63 received < 58.6 Gy × 2, and 95% of PTV54 received < 45.9 Gy × 2).

Overall, there were a total of 10 major violations in either contour or dosimetry in nine patients, 13.8% of the RT QA population. The major variations were two for unacceptable contours (GTVn × 1 and PTV70 × 1) and eight for dosimetry (mandible D1% > 72.1 Gy, Brachial plexus D1% > 68 Gy × 4, 95% PTV70 receiving less than 65.1 Gy × 2, 95% GTVp receiving less than 65.1 Gy).

#### RT QA of the patients with loco-regional failure

There were nine LRFs in the 182 treated patients: six regional, two local, and one loco-regional. Of these nine cases, the results of the RT QA associated with each case are tabulated in **Table 2**.

**Table 2:** Distribution of the RT QA processes for the loco-regional failure patients (*n* = 9).

	Yes	No
Benchmark case submitted	8	1
Institutional RT QA	5	4
Pre-treatment RT QA review	1	8
Post-treatment RT QA review	3	6

In these nine cases, four had undergone study RT QA and five had not. In the four cases that had undergone review, three were reviewed post-treatment and one was reviewed pre-treatment. The RT QA reviews in these patients had not shown any major protocol violations, and the second review (JC) was concordant in three cases (75%). The non-concordant case had originally been reviewed post-treatment as having no major protocol violations, but the second review assessment had major protocol violations in the contouring of GTVn and hence PTV70, and this had a significant probability of contributing to the regional failure. This regional recurrence was not resectable and the patent died of disease.

**Figure 3:**
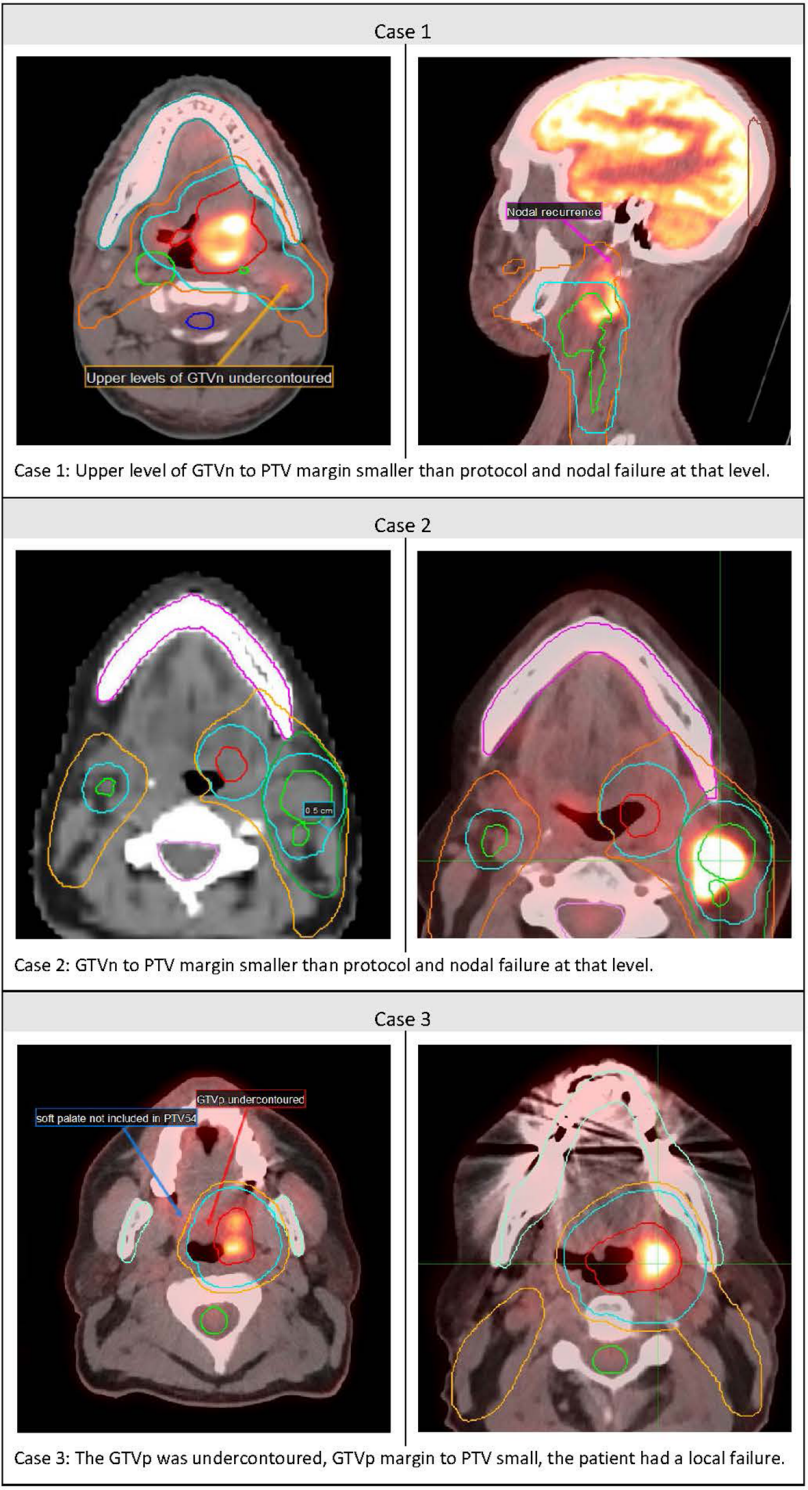
Cases of Major Protocol violations and loco-regional failure.

In the five cases not previously reviewed, RT QA revealed major protocol violations in three cases (60%), with a significant probability of contributing to local or regional failure (see **Figure 3**). In the first case, the GTVn was assessed as under contoured and hence the GTVn-to-PTV70 margin was too small. In addition, the margin from GTVp to PTV70 was 7 mm and the protocol recommended a minimum of 10 mm. This patient died of local and regional failure. In the second case, the margin from GTVn to PTV70 ranged from 4 to 6 mm, and this patient failed in the neck. They had a salvage neck dissection and remain alive with no evidence of disease. In the third case, the GTVp was assessed as under contoured with a subsequent close GTV-to-PTV margin, and this probably contributed to their local failure. They had surgical salvage and remain alive with no evidence of disease. In none of these cases were the less-than-10-mm GTV-to-TV margins related to anatomical boundaries.

## Discussion

The study represents the most detailed report of any RT QA process in an HNC study in the IMRT era.

Over a third of the study patients had comprehensive RT QA. Within that group, 86% had no major protocol violations, and if brachial plexus was omitted as a Priority 1 structure (four cases in three patients), then 90% would have had no major treatment violations.

Thus, the question remains—what items should be included as a minimum for adequate RTQA? As mentioned, the main purpose of HNC RT QA is to optimise cancer control probability and minimise the risk of serious late treatment toxicity. Hence, in terms of items to be assessed, it is not controversial to include GTVp, GTVn, and PTV70 and spinal cord as “Priority 1” structures in oropharyngeal cancer RT QA. However, inclusion of the mandible and brachial plexus, and the maximum doses that constitute a major protocol violation is more controversial. We believed that they were worthy of inclusion because mandibular osteoradionecrosis and brachial plexopathy are serious late treatment toxicities that cause considerable patient morbidity.

There is considerable variation in dosimetry criteria used for RT QA in different protocols. **Table 3** compares the RT QA criteria in the protocols of TROG 12.01 and RTOG 1016 (3). It is important to remember that the RTOG 1016 protocol was for 70 Gy in 35 fractions over 6 weeks, with PTV2 56 Gy and PTV3 50 Gy, whereas TROG 12.01 prescribed 70 Gy in 35 fractions over 7 weeks with PTV2 63 Gy and PTV3 54 Gy. However, if we compare “Priority 1 structures”, in the RTOG 1016 trial, there was no major protocol violation ascribed to GTV coverage, whereas in the TROG 12.01 trial, the GTV had to receive a minimum of 66.5 Gy. For PTV70, the major deviation was 95% receiving <65.1 Gy in the TROG 12.01, but in RTOG 1016, it was only if <63 Gy. Spinal cord doses were also recorded differently. In TROG 12.01, if the maximum point dose to the spinal cord was >46.4 Gy, it was a major violation, whereas in RTOG 1016, it was >50 Gy, which was considered a major violation.

**Table 3:** Comparisons of Items included in HNC RT QA trial protocols.

Item	TROG 12.01	RTOG 1016	Major Deviations TROG 12.01	Major Deviations RTOG 1016
GTV	Mandatory	Mandatory	Minimum dose > 66.5 Gy	Not stated
GTV-PTV margin	Mandatory	Mandatory	10–15 mm	10–25 mm
PTV70	Mandatory	Mandatory	95% PTV70 < 65.1 Gy	95% PTV70 < 63 Gy
PTVid*	Mandatory	Mandatory	95% PTVid < 58.6 Gy	95% PTVid < 45 Gy
PTVld#	Mandatory	Mandatory	95% PTVld < 45.9 Gy	95% PTVld < 40 Gy
Spinal cord PRV	Optional	Mandatory	Not stated	>52 Gy
Spinal cord	Mandatory	Mandatory	> 46.4 Gy	>50 Gy
Brain stem PRV	Optional	Mandatory	Not stated	>52 Gy
Mandible	Mandatory	Optional	>72 Gy	>66 Gy
Brach Plex	Mandatory	Not required	>68 Gy	Not stated
Max dose in PTV1			>77 Gy	>82 Gy
			Recommended doses	Recommended doses
Parotids	Mandatory	Mandatory	Mean < 26 Gy	Mean < 26 Gy
Pharynx	Mandatory	Optional	Mean < 63 Gy	Uninvolved mean < 45 Gy
Glottis	Mandatory	Optional	Mean < 45 Gy	Mean < 20 Gy
SMG	Mandatory	Optional	Mean < 39 Gy	Mean < 39 Gy
Oral cavity	Mandatory	Optional	Mean < 42 Gy	Uninvolved mean < 30 Gy
Lips	Optional	Optional	Not stated	Mean < 20 Gy
Cervical oesoph	Optional	Optional	Not stated	Mean < 30 Gy

*PTVid, planning target volume intermediate dose; #PTVld,– planning target volume low dose.

The brachial plexus D1% maximum dose was a major violation if greater than 68 Gy in TROG 12.01, but it was not mandated in RTOG 1016. The maximum mandible dose in TROG 12.01 was 70 Gy, and D1% maximum dose > 72 Gy was a major violation. In RTOG 1016, it was recommended the maximum dose be less than 66 Gy, but it did not seem to be a major violation. Interestingly the maximum dose allowed within PTV1 in TROG 12.01 was D2% 77 Gy and any higher was a major violation, whereas in RTOG 1016, a PTV1 hot spot was accepted up to 82 Gy. Thus, two experienced HNC trial groups, within the same disease subsite, demonstrate significant differences for structures and dosimetric constraints recorded as major protocol violations. Hence, that needs to be standardised by a consensus statement from major HNC research groups for future studies.

Regarding the number and timing of cases be reviewed, when first planning the RT QA for this study, we had thought completion of the benchmark case may be the most important component of the RT QA process. Successful completion of the benchmark case would be the best way to ensure that all had read and adhered to the study RT protocol prior to entering patients on the study.

However, eight of the nine cases of LRF had had a benchmarking case performed by that clinician; thus, this suggests that it may not be that helpful in reducing specific RT protocol violations.

A significant proportion (3/9, 33%) of the LRF cases had had post-treatment review, but this is of limited value as the patient has completed treatment and any protocol violations cannot be adjusted. It is helpful as a general overview of the quality of the RT delivered, but not for reducing adverse outcomes in any particular patient.

Perhaps the greatest concern was that eight of the nine patients with LRF had not had pre-treatment review. This is clearly the timing that allows corrections before treatment (as per the Peters et al. study) and hence most directly correlates with better oncological outcomes. Thus, pre-RT QA is the area that deserves greater concentration of resources. Pre-treatment review in the TROG 12.01 study resulted in a 40% reduction in Priority 1 major violations, given that three of five cases with major violations in Priority 1 structures were corrected prior to treatment. Theoretically, if the nine patients with LRF had all undergone pre-treatment RT QA review, and the major protocol violations in Priority 1 structures had been corrected, then the LRF rate could *potentially* have been reduced by 4, i.e., from 9 to 5, so a reduction in the LRF rate from 9/182 (4.9%) to 5/182 (2.7%), or a halving of the rate of LRF.

The financial cost of RT QA is not insignificant but may vary greatly between different countries. Dosimetry recalculations and dosimetric protocol violations can be recorded automatically via programmes such as CQMS (central quality management system). What cannot currently be automated is the review of GTV and PTV contours. It is possible that in the future, artificial intelligence could be helpful in this area. Currently, the average time taken for RT QA review of imaging and contours by an independent HNRO is approximately 20 min per case (4), or approximately 60 AUD. Quite apart from the emotional cost of salvaging an LRF, the financial cost is high. There are no Australian figures for the cost of managing LRF, but American and European studies suggest that it is in the order of 30,000 AUD (5, 6). In fact, in the TROG 12.01 study, all the HNRO RTQA was performed on an honorary basis. However, if not, the approximate costing of contour reviews for all 182 patients’ pre-treatment would have been approximately 11,000 AUD, or roughly a third of managing a single recurrence.

Finally, what proportion of HNC patients need to undergo RTQA for optimal results? There are no data to answer that question. To date, percentages used range around the 10% mark (7), but this is a pragmatic response to available resources rather than a scientific or financial costing of relative benefit.

Ideally, one would review all cases and see if an algorithm could be formulated to determine the optimal percentage of cases needed to undergo pre-treatment RT QA for optimal or most efficient detection of major protocol violations.

## Conclusions

RT QA is important for the optimal management of HNC. This study reinforces the point that pre-treatment peer review with formal RT QA and feedback to the treating HNRO offers the highest likelihood of reducing major protocol violations and improving patient outcomes. There needs to be consensus as to the items to be included in RT QA, but GTV, PTVs, and critical OAR are a good starting point. The percentage of cases that should undergo such review requires further study. Nevertheless, we need to arrest the drift of RT QA being performed post-treatment. Pre-treatment RT QA needs to be a standard procedure during the treatment planning stage and deserves appropriate allocation of resources for the optimal management of HNC patients.

## Data availability statement

The raw data supporting the conclusions of this article will be made available by the authors, without undue reservation.

## Ethics statement

The studies involving humans were approved by Peter MacCallum Cancer Center, Melbourne, Australia. The studies were conducted in accordance with the local legislation and institutional requirements. Written informed consent for participation was not required from the participants or the participants’ legal guardians/next of kin in accordance with the national legislation and institutional requirements.

## Author contributions

JC: Conceptualization, Writing – original draft, Writing – review & editing. AM: Data curation, Formal Analysis, Writing – review & editing. LK: Writing – review & editing. CW: Writing – review & editing. TF: Writing – review & editing. CLin: Writing – review & editing. SP: Writing – review & editing. CLiu: Writing – review & editing. MR: Writing – review & editing. AS: Writing – review & editing. LM: Writing – review & editing. DW: Writing – review & editing. AT: Writing – review & editing. DR: Writing – review & editing.
